# Intraoperative C-arm CBCT versus early postoperative MRI for the assessment of fracture reduction and osteosynthesis material positioning in cranio-maxillofacial trauma surgery

**DOI:** 10.1007/s10006-026-01588-8

**Published:** 2026-06-26

**Authors:** Adib Al-Haj Husain, Sameena Sandhu, Egon Burian, Marc M. Precht, Stefan Sommer, Thomas Frauenfelder, Maximilian Eberhard Hermann Wagner, Harald Essig

**Affiliations:** 1 Department of Cranio-Maxillofacial and Oral Surgery, University Hospital Zurich, University of Zurich, Rämistrasse 100, Zurich, 8091 Switzerland; 2 Department of Cranio-Maxillofacial Surgery, GROW School for Oncology and Reproduction, Maastricht University Medical Centre, Maastricht, The Netherlands; 3 Diagnostic and Interventional Radiology, University Hospital Zurich, University of Zurich, Zurich, Switzerland; 4 Swiss Center for Musculoskeletal Imaging (SCMI), Balgrist Campus, Zurich, Switzerland; 5 Swiss Innovation Hub, Siemens Healthineers International AG, Zurich, Switzerland

**Keywords:** (MeSH), Magnetic resonance imaging, Cone-beam computed tomography, Intraoperative imaging, Maxillofacial trauma, Maxillofacial surgery, CT-like magnetic resonance imaging

## Abstract

**Purpose:**

To compare intraoperative C-arm cone-beam computed tomography (CBCT) with dedicated in-house-optimized magnetic resonance imaging (MRI) protocols, including CT-like and metal artifact reduction sequences (MARS), using a novel 15-channel coil for the evaluation of fracture reduction, osteosynthesis material positioning, and the depiction of plates and screws.

**Methods:**

Fifteen patients (13 men, 2 women; median age, 32 years; range, 18–65 years) with acute cranio-maxillofacial fractures underwent surgical treatment with intraoperative CBCT, followed by early postoperative MRI within 36 h. MRI protocols included 3D ultrashort echo time (UTE), slice-encoding for metal artifact correction (SEMAC), and 3D double-echo steady-state (DESS). Two independent maxillofacial surgeons performed qualitative and quantitative evaluations of fracture reduction, osteosynthesis material positioning, and implant morphology using a 5-point visual scale (5 = best; 1 = non-assessable) and dimensional measurements. Descriptive statistics were calculated, and inter-reader agreement was assessed using weighted kappa (κ) and intraclass correlation coefficients (ICC).

**Results:**

UTE and SEMAC sequences provided excellent CT-like performance in visualizing fracture reduction and implant positioning (median 5, IQR 5–5 and 4–5, respectively), with minimal absolute differences in quantitative measurements and substantial to almost perfect inter-reader agreement (κ = 0.75-1.0; ICC = 0.89–0.99; all *p* < 0.001). In contrast, DESS demonstrated limited utility for assessing metallic implants due to pronounced signal voids and susceptibility artifacts.

**Conclusion:**

UTE and SEMAC provide high-quality, radiation-free imaging for evaluating fracture reduction and osteosynthesis material in cranio-maxillofacial trauma. These techniques demonstrate comparable diagnostic performance to intraoperative CBCT in a postoperative setting. They may serve as a radiation-free alternative for perioperative imaging in younger patients with anticipated repeated imaging needs, pending prospective intraoperative validation.

**Trial registration number:**

Swiss National Clinical Trials Portal: SNCTP000006343, ClinicalTrials.gov ID: NCT07012850, Registration date: May 2025.

## Introduction

Accurate fracture reduction and precise placement of osteosynthesis material are essential for achieving optimal functional and aesthetic outcomes in the surgical management of complex cranio-maxillofacial trauma. Intraoperative imaging has become an integral part of contemporary surgical workflows, allowing immediate verification of surgically relevant parameters and supporting adherence to preoperative surgical planning. By enabling timely intraoperative corrections, it may ultimately reduce the need for revision surgery while potentially improving postoperative outcomes [1, 2].

In oral and maxillofacial surgery, three-dimensional (3D) X-ray-based cone-beam computed tomography (CBCT) represents the primary modality for intraoperative imaging. C-arm CBCT has become widely established due to its high-resolution depiction of osseous structures, fast image acquisition, broad availability, and seamless integration into surgical workflows and the operating room environment [1, 2]. However, routine intraoperative use can be associated with several limitations, including repeated radiation exposure, often affecting a comparatively young patient population, increased procedural costs, and prolonged operative times [3, 4]. By contrast, radiation-free magnetic resonance imaging (MRI) is routinely used intraoperatively in other surgical disciplines [5, 6]; however, its limited hard tissue contrast and susceptibility to metal-induced artifacts have historically limited its applicability in perioperative cranio-maxillofacial interventions, particularly in cases involving extensive reconstructions [7].

Recent advances in MRI for dentomaxillofacial applications, including the development of specialized MR coils, novel MRI protocols that provide CT-like bone depiction, and advanced metal artifact reduction sequence (MARS) techniques, have substantially expanded its perioperative clinical utility in cranio-maxillofacial and oral surgery [8–10]. Studies investigating both pre- and postoperative trauma imaging have demonstrated promising CT-like diagnostic performance, enabling precise fracture and dislocation assessment while simultaneously providing high-resolution visualization of associated soft-tissue structures [11–14].

Although both modalities are increasingly accessible, comparative data on their effectiveness in evaluating fracture reduction and osteosynthesis material positioning in cranio-maxillofacial trauma surgery are lacking. Therefore, this prospective study aimed to compare intraoperative C-arm CBCT with early postoperative MRI acquired using dedicated, in-house-optimized protocols and a novel 15-channel coil to assess fracture reduction and osteosynthesis material positioning and delineation.

## Materials and methods

### Study design

This prospective comparative study enrolled patients with cranio-maxillofacial trauma who were treated as part of routine clinical care at the Department of Cranio-Maxillofacial and Oral Surgery at the University Hospital Zurich, Switzerland, between May and August 2025.

Patients were eligible for inclusion if they were (1) adults (≥ 18 years) (2), presented with acute cranio-maxillofacial trauma (3), underwent surgical management of trauma with intraoperative CBCT imaging, and were able to provide informed consent. Patients were excluded if they were pregnant, had contraindications to MRI, had relevant comorbidities likely to compromise image quality or compliance with imaging or study procedures, or were enrolled in other clinical studies with potential confounding effects on imaging outcomes.

All enrolled participants received intraoperative CBCT imaging, followed by postoperative MRI within 36 h after surgery. All examinations were conducted in accordance with standard clinical care pathways for trauma management and did not result in any additional radiation exposure beyond routine clinical imaging. Imaging procedures were performed by experienced surgeons and clinical and research personnel from the Departments of Cranio-Maxillofacial and Oral Surgery and Radiology at the University Hospital Zurich.

Participants in this study were also included in a separate study comparing preoperative imaging [14]; however, the intraoperative and early postoperative imaging datasets analyzed in the present study have not been previously evaluated or reported.

The study protocol was reviewed and approved by the Cantonal Ethics Commission of Zurich, Switzerland (approval number 2024–02307). All participants provided written informed consent prior to inclusion, and the study was conducted in full compliance with the principles of the Declaration of Helsinki and its subsequent revisions.

### Surgical intervention

Surgical management of cranio-maxillofacial trauma included the use of osteosynthesis materials composed of titanium or titanium alloys (all KLS Martin Group, Tuttlingen, Germany). Reconstruction strategies included patient-specific implants, standard titanium or titanium alloy plates and screws, and mesh constructs. The reconstruction material and technique were selected based on fracture location, defect size, and the surgeon’s evaluation of biomechanical needs, aiming to restore ideal anatomical structure and optimal functional outcomes.

### Image acquisition

#### Intraoperative CBCT acquisition

All study participants underwent intraoperative 3D imaging using an X-ray-based C-arm CBCT imaging system (OEC 3D mobile, GE HealthCare Technologies Inc., Chicago, USA) in accordance with the institution’s standard clinical workflow. The system provides high-resolution intraoperative imaging with a cubic field of view (FOV) of 19 cm × 19 cm × 19 cm and isotropic voxel dimensions of 0.3–0.6 mm, supporting multiplanar reconstructions, maximum intensity projection, and volume rendering [15].

#### Postoperative MRI acquisition

The same study participants underwent MRI examinations on a 3 Tesla MAGNETOM Vida^fit^ system (Siemens Healthineers, Forchheim, Germany) utilizing a specialized 15-channel coil (NORAS MRI Products, Hoechberg, Germany) to optimize image quality for the cranio-maxillofacial region. Three in-house optimized MRI sequences were obtained with sub-millimeter isotropic resolution: a research application 3D ultrashort echo time (UTE) prototype protocol, a slice-encoding for metal artifact correction (SEMAC) sequence, and a 3D double-echo steady-state (DESS) protocol. Sequence selection was based on published evidence demonstrating UTE’s CT-like depiction and robust artifact reduction, SEMAC’s effectiveness in mitigating metal-related artifacts, and DESS’s superior soft-tissue contrast [16–18]. Sequence parameters were as follows: UTE: repetition time (TR), 4.6 ms; echo time (TE), 0.04 ms; flip angle, 5°; bandwidth, 1184 Hz/Px; no fat suppression; matrix, 384 × 384 × 384; voxel size, 0.6 × 0.6 × 0.6 mm^3^; acquisition time, 3:05 min. SEMAC: TR, 3370 ms; TE, 39 ms; flip angle, 120°; bandwidth, 599 Hz/Px; no fat suppression; matrix, 288 × 202; voxel size, 0.5 × 0.5 × 3.0 mm^3^; acquisition time, 5:55 min. DESS: TR, 11.2 ms; TE, 4.21 ms; flip angle, 30°; bandwidth, 355 Hz/Px; fat suppression, water excitation normal; Phase encoding direction, R » L; matrix read/phase 104 × 104; total acceleration factor, 2; voxel size, 0.4 × 0.4 × 0.8 mm^3^; acquisition time, 6:41 min. Images were acquired in the axial or coronal planes and, when feasible, further processed using multiplanar reconstruction to generate additional orientations. For orbital and midface cases, multiplanar reconstructions were generated with reference to a standardised 3D coordinate system. This included paramedian oblique-sagittal reformats to enable reproducible side-by-side comparison between CBCT and MRI datasets. Figure presentations were aligned to identical anatomical reference coordinates across modalities, to the extent permitted by the respective acquisition geometries.

### Image analysis

Intraoperative CBCT and MRI datasets were saved in DICOM format and assessed using the institution’s Picture Archiving and Communication System (PACS) in DeepUnity Diagnost (v2.0.2.2, Dedalus HealthCare, Bonn, Germany) and Synedra View (version 24.0.0.3 (x64 Edition), Synedra Information Technologies GmbH, Innsbruck, Austria). Image assessments were independently performed by two maxillofacial surgeons with different levels of expertise: Reader A (S.S.), a board-certified attending with 14 years of clinical experience; and Reader B (A.A.H.), a resident with 5 years of experience. A training session was conducted to calibrate the observers and standardize the image evaluation process. To reduce potential bias, each reader was blinded to the other reader’s results and to the specific MRI protocol, and all images were reviewed in a randomized order. Each reader was allowed to adjust the window settings and zoom level as needed during image evaluation.

### Qualitative analysis

Evaluation of fracture reduction, the accuracy of osteosynthesis material placement, and the delineation of osteosynthesis plates and screws’ structural contours was performed.

Visualization of fracture reduction and osteosynthesis material positioning was evaluated using a modified 5-point visual analog scoring system [19, 20]. A score of 5 indicated excellent depiction, with clear, unambiguous visualization of fracture reduction and implant positioning. A score of 4 represented a good depiction with minor limitations that did not meaningfully affect fracture reduction and implant positioning assessment. A score of 3 corresponded to adequate depiction with noticeable limitations that clearly affected the visualization of fracture reduction and implant positioning. A score of 2 indicated poor depiction, with markedly restricted visualization of fracture reduction and implant positioning. A score of 1 indicated a non-assessable depiction, in which reliable assessment of fracture reduction and implant positioning was not possible.

A modified 5-point visual analog rating scale was also used to evaluate the morphology of osteosynthesis plates and screws [20]. A score of 5 reflected excellent visualization, characterized by a complete and sharply defined depiction of plate contours and all screw components. A score of 4 indicated good visualization, with clear representation of plates and screws and only minimal loss of sharpness or detail. A score of 3 indicated adequate visualization, in which plate and screw contours remained identifiable despite a moderate reduction in definition. A score of 2 corresponded to poor visualization, with only partial visibility of plate and screw contours and substantial loss of sharpness. A score of 1 indicated non-assessable visualization, with plate and screw contours indistinct and not reliably evaluable.

### Quantitative analysis

Quantitative measurements were performed by both readers on CBCT and MRI datasets, determining postoperative residual fracture displacement, if present, as the maximal interfragmentary gap or step-off at the fracture site and expressed in millimeters. Additionally, measurements are provided for osteosynthesis plates’ thickness and length, as well as screw length and diameter, each recorded at their maximum dimension in millimeters. These values were subsequently used for protocol-specific and inter-modality comparisons, following a modified evaluation approach adapted from previously published methodology [11, 20].

### Statistical analysis

Descriptive statistics were computed for all variables, including mean, median, interquartile range (IQR), and standard deviation. Agreement between the two readers was quantified using weighted kappa coefficients for qualitative data and interpreted according to established thresholds [21]: poor (< 0), slight (0.00–0.20), fair (0.21–0.40), moderate (0.41–0.60), substantial (0.61–0.80), and almost perfect (0.81–1.00). Quantitative parameters were evaluated using the Intraclass Correlation Coefficient (ICC) [21]. ICC values were interpreted as follows: poor (< 0.5), moderate (0.5–0.75), good (0.75–0.9), and excellent (> 0.9). Intermodal differences in quantitative parameters were expressed as absolute and relative differences. A two-sided significance level of α = 0.05 was used for all statistical tests.

## Results

Fifteen patients (13 men and 2 women; mean age, 34.1 ± 13.6 years; median age, 32 years; range, 18–65 years) presenting with cranio-maxillofacial fractures of the mandible (32%), midface (25%), orbit (25%), nasal fractures (11%), and frontal sinus (4%) were included in this trial. The interval between intraoperative CBCT and postoperative MRI ranged from 5.9 to 33.5 h, with a mean of 27.1 ± 10.5 h and a median of 29.1 h. Two independent observers evaluated all 15 intraoperative CBCT scans and 44 corresponding MRI reconstructions, noting that one MRI sequence was unavailable due to incomplete acquisition.

### Qualitative results

Visualization of fracture reduction and osteosynthesis material positioning was rated as excellent to perfect, with CBCT achieving perfect scores (median 5, IQR 5–5) and UTE and SEMAC achieving excellent scores (median 5, IQR 5–5 and 4–5, respectively). In contrast, DESS sequences provided poor visualization (median 2, IQR 2–2).

CBCT and UTE provided the most effective assessment of osteosynthesis plates and screws, with assessments ranging from excellent to good, while DESS sequences were mostly non-assessable for both screws and plates, yielding median rating scores of 1–2 (IQR 1–2 and 1–1, respectively).

Inter-reader agreement was substantial to almost perfect across all evaluated parameters and imaging protocols (κ = 0.75–1.0, all *p* < 0.001).

**Fig. 1:**
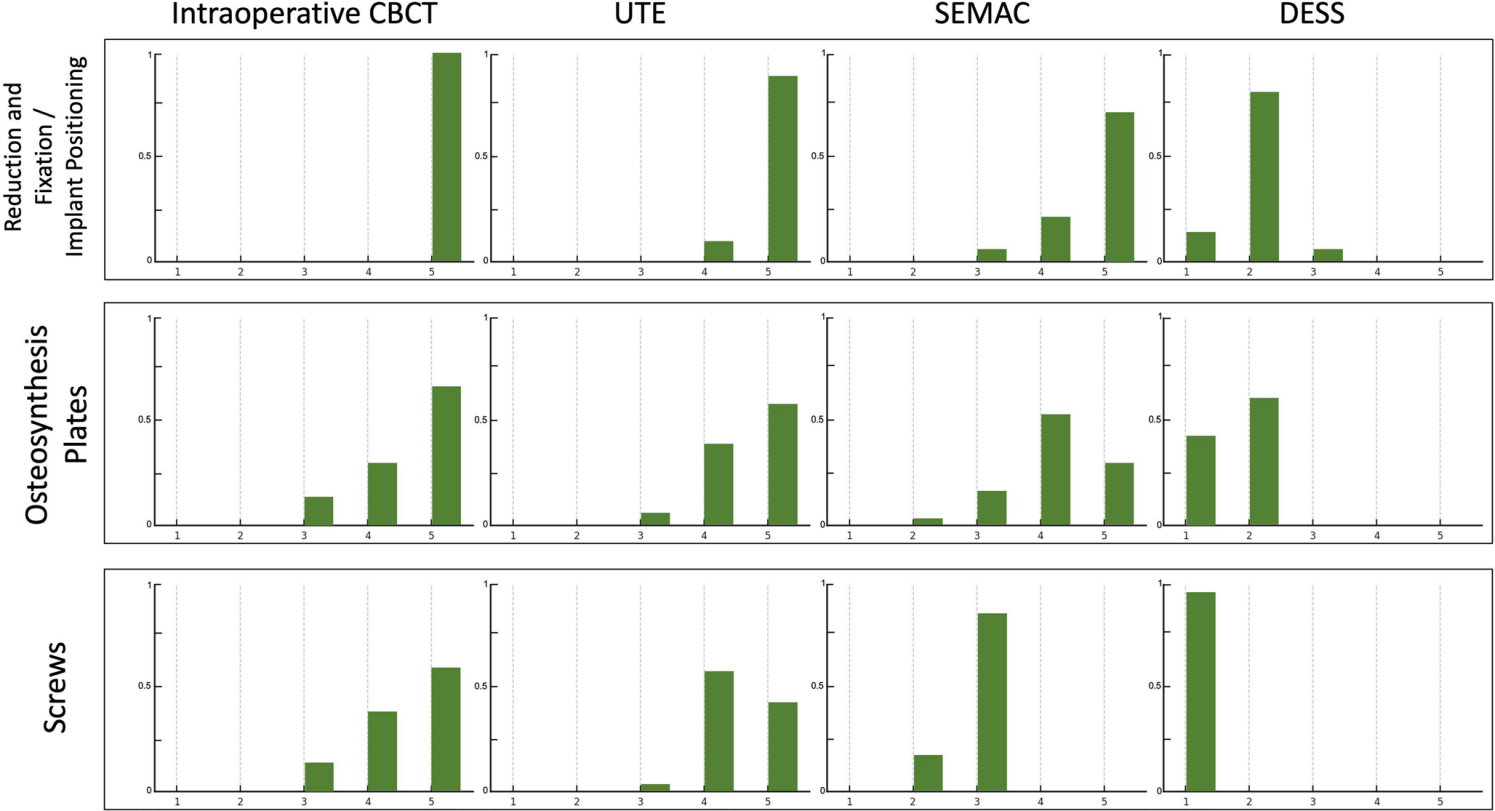
Distribution of qualitative visual grading scores for fracture reduction and fixation, and the depiction of osteosynthesis plates and screws, shown for intraoperative CBCT and each MRI protocol. Scores were rated on a 5-point scale, with 5 representing the most favorable visualization and 1 the least favorable. **CBCT**: Cone-beam computed tomography; **UTE**: 3D ultrashort echo time;** SEMAC: **Slice-encoding for metal artifact correction sequence;** DESS**: 3D double-echo steady-state

A detailed summary of the qualitative assessment is presented in Table 1, while the corresponding frequency distribution of visual scores is shown in Fig. 1.

**Table 1:** Two independent readers (Reader A: attending physician; Reader B: resident) performed a qualitative assessment of fracture reduction and osteosynthesis material positioning, and the clarity of structural contours of plates and screws using a modified 5-point visual analog scale (1 = non-assessable, 5 = excellent)

	Imaging Protocol	Reader A	Reader B	Inter-Reader agreement (κ) and 95% confidence interval (CI)
Fracture reduction and osteosynthesis materials positioning	CBCT UTE SEMAC DESS	5 (5–5) 5 (5–5) 5 (4–5) 2 (2–2)	5 (5–5) 5 (5–5) 5 (4–5) 2 (2–2)	1.0 (1.0–1.0); *p* < 0.001 1.0 (1.0–1.0); *p* < 0.001 0.88 (0.64–1.0); *p* < 0.001 0.77 (0.38–1.0); *p* < 0.001
Osteosynthesis plates	CBCT UTE SEMAC DESS	5 (4–5) 5 (4–5) 4 (4–5) 2 (1–2)	5 (4–5) 5 (4–5) 4 (4–5) 2 (1–2)	1.0 (1.0–1.0); *p* < 0.001 0.89 (0.68–1.0); *p* < 0.001 0.83 (0.65–1.0); *p* < 0.001 0.76 (0.47–1.0); *p* < 0.001
Screws	CBCT UTE SEMAC DESS	4 (4–5) 4 (3–4) 3 (3–3) 1 (1–1)	4 (4–5) 4 (3–4) 3 (3–3) 1 (1–1)	0.89 (0.67–1.0); *p* < 0.001 0.75 (0.46–1.0); *p* < 0.001 0.76 (0.42–1.0); *p* < 0.001 1.0 (1.0–1.0); *p* < 0.001

**CBCT**: Cone-beam computed tomography; **UTE**: 3D ultrashort echo time; **SEMAC**: Slice-encoding for metal artifact correction sequence; **DESS**: 3D double-echo steady-state

Results are reported as medians with interquartile ranges. Inter-reader agreement was evaluated using weighted kappa (κ) statistics

### Quantitative results

Quantitative measurements of fracture gaps, plate dimensions, and screw dimensions demonstrated high concordance between intraoperative CBCT and UTE and SEMAC MRI sequences. Absolute differences in fracture gap size were small for UTE (0.32 ± 0.24 mm) and SEMAC (0.47 ± 0.30 mm), but substantially higher for DESS (1.43 ± 0.73 mm). Similarly, plate thickness and length, as well as screw length and diameter, were more accurately assessed with UTE and SEMAC sequences. In contrast, DESS measurements were limited by poor visualization and metal-induced signal voids, resulting in overestimation of dimensions, particularly plate thickness (3.0 ± 1.1 mm; relative difference: 220 ± 100%).

**Fig. 2:**
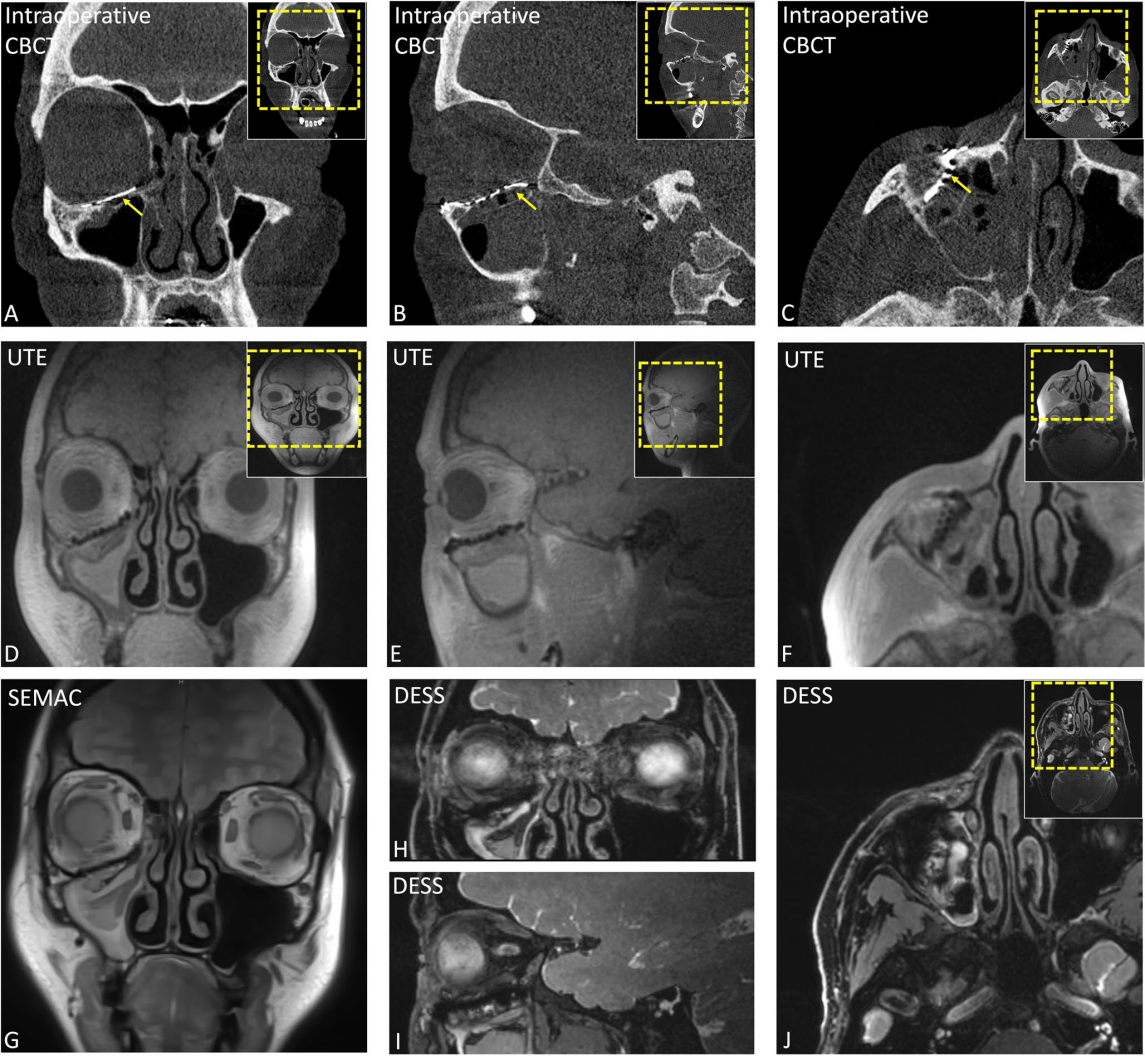
Right orbital floor fracture in a 42-year-old female patient following a bicycle accident, reconstructed with a patient-specific titanium orbital implant (IPS Implants® Midface Orbita, KLS Martin Group, Tuttlingen, Germany) (arrow) and fixed with a 4 mm screw. Intraoperative imaging was performed using C-arm CBCT (**A**: coronal, **B**: sagittal, **C**: axial), and MRI-based visualization was obtained with UTE (**D**-**F**), SEMAC (**G**), and DESS (**H**-**J**) sequences. **CBCT**: Cone-beam computed tomography; **UTE**: 3D ultrashort echo time;** SEMAC: **Slice-encoding for metal artifact correction sequence;** DESS**: 3D double-echo steady-state

**Fig. 3:**
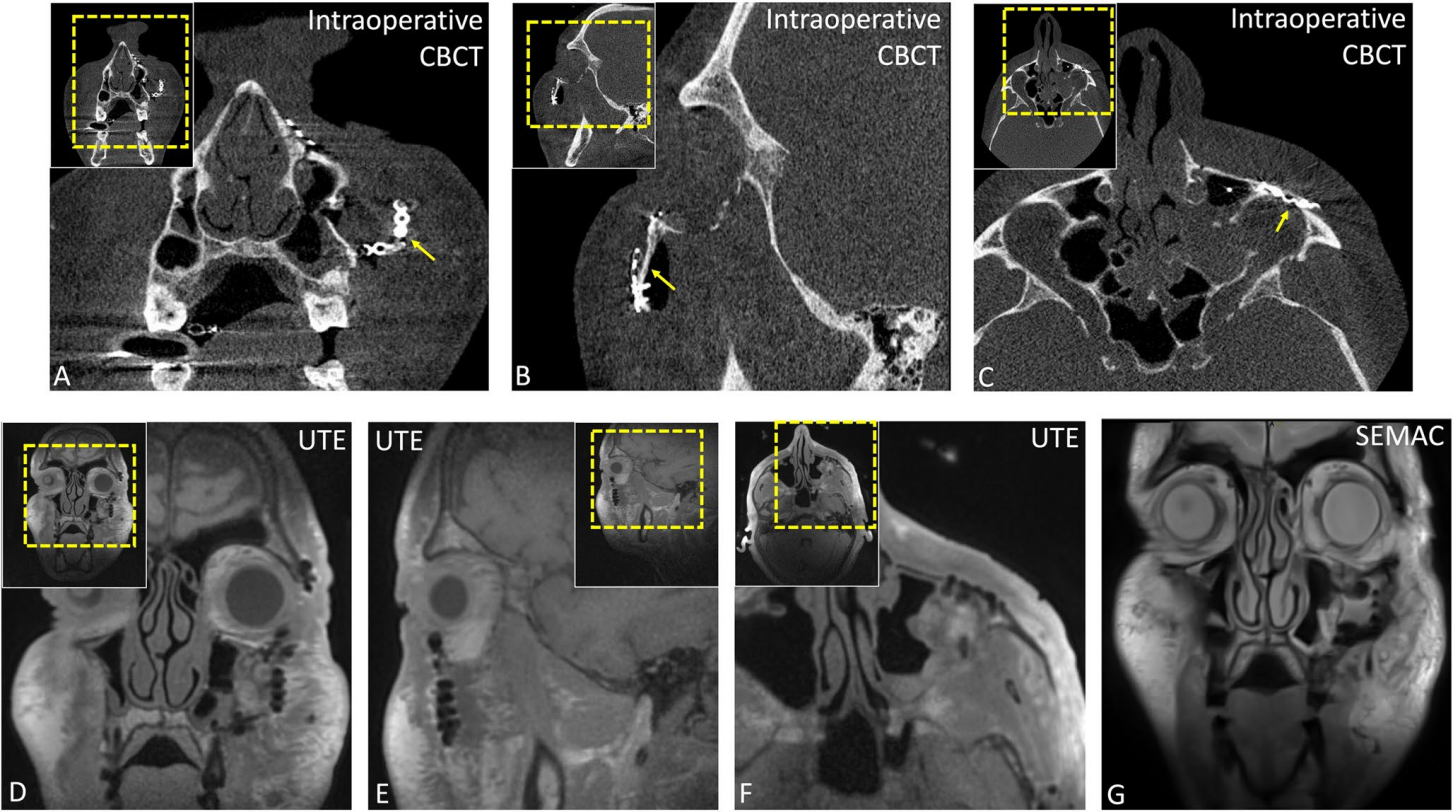
Multiple fractures of the left midface. Open reduction and internal fixation (ORIF) of the left zygomatic fracture, including the zygomatic arch attachment (arrow). Intraoperative imaging was performed using X-ray-based C-arm CBCT (**A**-**C**), and radiation-free, CT-like depiction was obtained with MRI using UTE (**D**-**F**), and visualization with the SEMAC (**G**) sequence. **CBCT**: Cone-beam computed tomography; **UTE**: 3D ultrashort echo time;** SEMAC: **Slice-encoding for metal artifact correction sequence;** DESS**: 3D double-echo steady-state

**Fig. 4:**
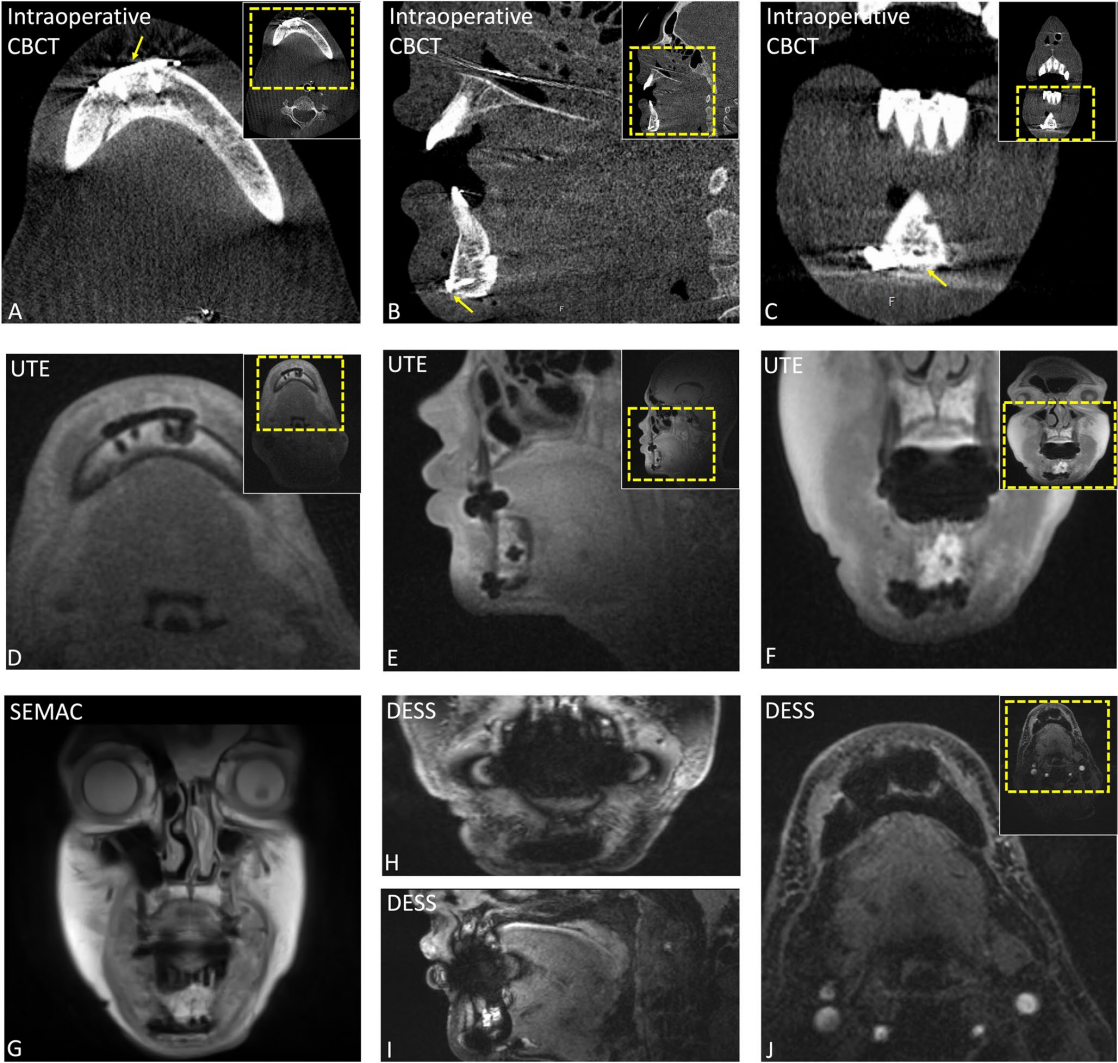
Management of a paramedian mandibular fracture using osteosynthesis plates and lag screws. Intraoperative CBCT images (**A**: coronal, **B**: sagittal, **C**: axial; arrows indicate osteosynthesis material). MRI-based visualization with UTE (**D**: coronal, **E**: sagittal, **F**: axial) and SEMAC (**G**) demonstrates signal voids caused by the osteosynthesis material, whereas fracture reduction and osteosynthesis material are poorly assessable on DESS images (**H**-**J**). **CBCT**: Cone-beam computed tomography; **UTE**: 3D ultrashort echo time;** SEMAC: **Slice-encoding for metal artifact correction sequence;** DESS**: 3D double-echo steady-state

Inter-reader reliability was excellent for all quantitative parameters across CBCT and MRI sequences (ICC 0.89–0.99, all *p* < 0.001) (Table 2; Figs. 2, 3 and 4).

**Table 2:** Comparison of MRI measurements with CBCT

	Imaging Protocol	Absolute Difference from CBCT (mean ± SD; in mm)	Relative Difference from CBCT (mean ± SD, as percentage (%))	Inter-Reader Agreement (ICC, 95% CI)
Fracture gap size	UTE SEMAC DESS	0.32 ± 0.24 0.47 ± 0.3 1.43 ± 0.73	23.4 ± 23.2 28 ± 20 88 ± 44	0.89 (0.65–0.97); *p* < 0.001 0.93 (0.79–0.98); *p* < 0.001 0.90 (0.68–0.99); *p* < 0.001
Plate thickness	UTE SEMAC DESS	1.0 ± 0.5 1.1 ± 0.5 3.0 ± 1.1	77 ± 63 91 ± 43 220 ± 100	0.92 (0.77–0.97); *p* < 0.001 0.93 (0.79–0.98); *p* < 0.001 0.96 (0.88–0.99); *p* < 0.001
Plate length	UTE SEMAC DESS	2.2 ± 1.8 2.8 ± 1.3 4.0 ± 2.2	8.8 ± 16.5 8.1 ± 5 12 ± 10	0.97 (0.91–0.99); *p* < 0.001 0.98 (0.96–0.99); *p* < 0.001 0.98 (0.93–0.99); *p* < 0.001
Screw length	UTE SEMAC DESS	1.5 ± 0.6 1.4 ± 0.7 3.6 ± 1.3	27 ± 21 27 ± 15 78 ± 41	0.99 (0.99–0.99); *p* < 0.001 0.99 (0.98–0.99); *p* < 0.001 0.99 (0.96–0.99); *p* < 0.001
Screw diameter	UTE SEMAC DESS	1.1 ± 0.6 1.3 ± 0.5 2.7 ± 1.3	57 ± 41 87 ± 38 150 ± 60	0.96 (0.89–0.98); *p* < 0.001 0.91 (0.73–0.97); *p* < 0.001 0.98 (0.94–0.99); *p* < 0.001

**CBCT**: Cone-beam computed tomography; **UTE**: 3D ultrashort echo time; **SEMAC**: Slice-encoding for metal artifact correction sequence; **DESS**: 3D double-echo steady-state

Quantitative analyses were performed to determine absolute and relative differences (reported in millimeters and percentages) and inter-reader agreement (Intraclass Correlation Coefficients (ICC) with 95% CI) are shown for fracture gap size, plate thickness and length, and screw length and diameter across UTE, SEMAC, and DESS sequences

## Discussion

This prospective comparative study demonstrates that dedicated MRI sequences, when combined with a novel 15-channel coil, provide high-quality, CT-like imaging for evaluating fracture reduction and osteosynthesis material positioning in cranio-maxillofacial trauma surgery. Both UTE and SEMAC sequences reliably depicted fractures and implants, with good to excellent qualitative ratings and minimal differences compared with intraoperative CBCT. These findings suggest that MRI may serve as a reliable, radiation-free alternative to ionizing intraoperative imaging in selected cases.

To the authors’ knowledge, this is the first study to directly compare dedicated MRI sequences with intraoperative CBCT in cranio-maxillofacial trauma surgery. In line with previous research on CT-like MRI sequences in musculoskeletal [22, 23] and craniofacial applications [24, 25], our results further confirm that 3D Gradient Echo (GRE)-based UTE and MARS, such as SEMAC, enable accurate, simultaneous depiction of osseous and soft-tissue structures [7, 16, 26], while also offering detailed assessment of fracture reduction and osteosynthesis material positioning. In our study setting, where patient movement and complex postoperative tissue environments are unavoidable, UTE and SEMAC sequences consistently provided excellent image quality and were robust against metal-induced susceptibility artifacts around cranio-maxillofacial implants, thereby supporting and extending the findings from earlier ex vivo and phantom studies [17, 27, 28]. Compared with the clinical intraoperative standard CBCT, CT-like MRI exhibits inherent limitations in depicting the fine morphology and contours of osteosynthesis materials, particularly screw geometry, as evaluation relies on indirect visualization through signal voids rather than direct structural representation. While the GRE-based DESS protocol provides superior soft-tissue contrast, it proved largely unsuitable for the assessment of metallic implants due to pronounced signal voids and susceptibility-related artifacts. These limitations are mainly attributable to the interaction between metal-induced magnetic field inhomogeneities and the protocol-specific signal characteristics of the DESS sequence [29]. Consequently, implant dimensions were frequently overestimated, resulting in reduced visibility of plates and screws, emphasizing the importance of careful, sequence-specific selection when applying MRI in the perioperative setting of cranio-maxillofacial trauma.

Quantitative analysis confirmed the reliability of UTE and SEMAC sequences, showing minimal differences in fracture gap measurements and plate and screw dimensions compared with CBCT [14, 17]. Inter-reader agreement was consistently high for both qualitative and quantitative parameter assessments, underscoring the reproducibility of these MRI protocols and supporting their potential implementation into standard intraoperative workflows.

From a clinical perspective, the complementary strengths of UTE and SEMAC MRI provide an optimal balance between effectively reducing metal-induced artifacts and reliably visualizing surgically relevant parameters. The minimal absolute quantitative differences compared with CBCT indicate comparable diagnostic performance in a postoperative setting, while offering the added advantage of avoiding ionizing radiation, an aspect that is particularly relevant in younger patient populations who may otherwise be exposed to repeated X-ray-based imaging.

However, several considerations must be taken into account when implementing radiation-free intraoperative imaging. A key limitation of MRI relates to the type of osteosynthesis material used. In this study, predominantly titanium implants were used, which are associated with low magnetic susceptibility and, consequently, generate fewer artifacts than ferromagnetic materials [29]. Additionally, implant composition, geometric complexity, and proximity to adjacent metallic structures may still lead to local magnetic field distortions and compromised visualization of the surgical site. Furthermore, integrating MRI into the operating room environment requires specialized infrastructure and equipment, potentially increasing procedural costs, prolonging image acquisition, and extending overall operative time.

An important distinction must be emphasised regarding the translational scope of these findings. The central clinical advantage of intraoperative CBCT lies in its immediacy. It enables real-time identification and correction of inadequate fracture reduction or implant malposition before wound closure and prior to patient extubation. The MRI examinations in the present study were performed within 36 h after surgery. They therefore do not replicate this intraoperative feedback loop. Accordingly, the present findings should not be interpreted as establishing direct intraoperative equivalence between MRI and CBCT. Rather, they support a clearly defined clinical role for early postoperative MRI as a radiation-free follow-up modality. This is particularly relevant in younger patients in whom cumulative radiation exposure from repeated imaging is a concern. The findings also serve as a proof-of-concept basis for future prospective intraoperative validation. True intraoperative MRI integration in cranio-maxillofacial surgery remains technically and logistically demanding. It requires dedicated MRI-compatible operating room infrastructure that is not yet available in standard surgical environments. Future studies should prioritise workflow-adapted intraoperative acquisition protocols to determine whether the diagnostic performance demonstrated here can be translated to a genuine intraoperative setting.

This study has several limitations. First, the relatively small cohort of 15 patients limits the generalizability of the findings. No formal a priori power calculation was performed. This study was designed as a prospective feasibility and comparative pilot study. Based on the effect sizes observed, a definitive validation study would require an estimated cohort of at least 40 to 50 patients to achieve adequate statistical power for confirmatory conclusions. Second, CBCT was used as a reference standard; however, no independent gold standard (e.g., surgical exploration or histological correlation) was available. Therefore, this study should be interpreted as a comparative modality assessment rather than a validation study. Third, MRI was not performed intraoperatively but within 36 h after surgery, which may have introduced minor discrepancies. However, image quality could be expected to improve under intraoperative conditions, as patient motion is minimized during general anesthesia. Fourth, only a limited number of MRI techniques were evaluated, indicating the need for a broader assessment. Fifth, the DESS sequence consistently underperformed in both qualitative and quantitative assessments. Pronounced susceptibility artifacts rendered implant evaluation largely non-assessable. Its routine inclusion in perioperative cranio-maxillofacial imaging protocols is therefore not recommended when titanium osteosynthesis material is present. UTE and SEMAC should be considered the sequences of choice. Future studies should therefore focus on implementing these protocols intraoperatively, optimizing workflows to reduce acquisition times, and validating these results in larger patient cohorts.

## Conclusion

This study demonstrates the feasibility and accuracy of CT-like MRI sequences and metal artifact reduction techniques for assessing fracture reduction and osteosynthesis material positioning in cranio-maxillofacial trauma surgery. In selected cases, UTE and SEMAC MRI provide a reliable, radiation-free alternative to CBCT. With further intraoperative validation, these techniques have the potential to enable fully radiation-free perioperative imaging workflows, allowing real-time surgical assessment without ionizing radiation exposure while simultaneously offering superior soft-tissue contrast compared with X-ray-based modalities.

## Data Availability

The datasets analyzed during the current study are available from the corresponding author on reasonable request.
